# Opening of the Royal Victoria-Infirmary, Newcastle

**Published:** 1906-07-21

**Authors:** 


### OPENING OF THE ROYAL VICTORIA INFIRMARY, NEWCASTLE.

On Wednesday, 11th inst., the King and Queen visited Newcastle from Alnwick Castle for the performance of various public functions. The weather was wet, but the town was profusely decorated, and their Majesties were welcomed by large crowds. At the Central Station their Majesties were received by the Mayor and Corporation, and in acknowledging an address of welcome the King intimated that the title of Lord Mayor would be conferred on the Mayor. A procession was then formed, and drove through the streets to Armstrong College, which the King formally opened. The ceremony here was all under substantial cover, but the infirmary was opened in a temporary pavilion fashioned from canvas in green and white, and having a dais upon which their Majesties were to stand. Here some three thousand ladies and gentlemen were gathered. The roof held water for a while, but when once the wet had penetrated the flimsy protection it poured into the pavilion in a great stream. The dais even was deluged in parts, but by the time their Majesties had arrived there was a temporary improvement in the weather.

Their Majesties were received at the entrance by the President, Dean Kitchin. On leaving the College their Majesties proceeded to the Royal Victoria Infirmary, and were received at the entrance by Lord Armstrong (Chairman of the House Committee), and also by Sir Riley Lord (Chairman of the Building Committee) and Sir George H. Philipson (Vice-Chairman of the House and Building Committees), who had the honour of being presented to the King and Queen by Lord Armstrong.

Their Majesties were conducted to the dais in the Royal pavilion, where the following had the honour of being presented to the King and Queen by Lord Armstrong: Dr. G. H. Hume (Chairman of the Furnishing Committee), Dr. D. Drummond (Chairman of the Hon. Staff), Mr. J. E. Woods (Treasurer), Mr. W. J. Sanderson (Chairman of the Administration Committee), Mr. C. Irwin (Chairman of the Contracts Sub-Committee), Mr. J. Mitchell (representative workman), Mr. H. R. Bailey (representative of the Cooperative Societies), Mr. T. Buckham (representative workman), and the joint architects (Mr. W. L. Newcombe and Mr. H. P. Adams).

An address was presented from the governing body by Lord Armstrong. In it they reminded his Majesty that he had laid the foundation-stone of the Infirmary on June 20, 1900, in the name of Queen Victoria. They asked their Majesties' permission to name one of the wards King Edward VII. Ward and another Queen Alexandra Ward.

The King, speaking in a voice perfectly audible throughout the room, said in reply :—

" It gives the Queen and myself great pleasure to be here to-day to open this fine building which bears the name of Queen Victoria, my beloved mother. We well remember our previous visit to this ancient city to lay the foundation-stone of the Royal Victoria Infirmary, and it is a sincere gratification to us to see the completion of this noble institution, which will be an abiding memorial of those munificent donors who have contributed so largely to its erection. It is a satisfaction to us also to know that the workers have given so generously towards the endowment of the Infirmary, from which they will secure benefits which are of the greatest value in a city where there are many industries involving risks to life and health. It will ever be our desire to do all that in us lies to assist institutions of which this infirmary is so fine an example, and to support every movement for the relief of the large class of persons suffering from disease who have no means for securing proper medical attention. We pray that the springs of charity will always flow abundantly in the furtherance of the beneficent work of alleviating distress and allaying suffering, and it is our earnest hope that this splendidly equipped building, where the services of skilled doctors and efficient nurses will be at all times available, will for many years to come be the means of restoring to health those whose career of usefulness has been interrupted by accident or illness. The good work done by Dr. Thomas Oliver, physician to the Infirmary, in connection with dangerous diseases is widely known and appreciated. The Queen and I willingly agree to the proposal that two wards be named Edward VII. Ward and Queen Alexandra Ward respectively. I thank you sincerely on behalf of the Queen and myself for your good wishes for our welfare, and I pray that the Divine blessing may attend your efforts in the fight against suffering and disease."

Their Majesties inspected some of the wards and before leaving the Infirmary the King accepted a copy of Dr. G. H. Hume's book, "The History of the Newcastle Infirmary," which is issued with the authority of the House Committee. The proceeds of the sale of this book will go to assist the Infirmary funds. Afterwards the King and Queen passed to the front of the building, where the statue of Queen Victoria had been placed. This the King unveiled.

### Description of Building.

We propose to defer our full description of this hospital as from a practical point of view—the only standpoint that is serviceable to our readers—it is not possible to adequately describe a building until it is completed. The following, however, will give a general idea of the building.

Only one ward was finished for inspection and we saw no other departments except the general kitchen and ward-maids' cubicles.

The new infirmary occupies a magnificent site, in an open space. The architects have made the best use of the ground, and the various departments are conveniently placed.

The ward open for inspection has twenty-four beds. In each ward there are small inspection windows from the ward kitchen and nurses' duty-room, these project on the face of the wall in the ward; they will be apt to collect dust and are too small to be of any real practical value, besides they are not necessary, as a ward with twenty-four beds should never be left without a nurse.

At the end of the ward are sanitary and bath-room towers, these have cut off passages dividing them from the ward with glass louvred windows, these glass louvres will be difficult to keep clean and will be very easily broken.

The bath-room contains one bath and two wash-hand basins, one in each corner of the bath-room. The basins would be better in another compartment as they cannot be used at the same time as the bath.

The other sanitary tower has two w.c.s with a wood partition about 8 ft. high dividing them; in this apartment there is also a sink and bed-pan washer. The w.c.s should be divided by a brick partition and be quite separate, a wooden screen is not satisfactory.

In the linen-rooms next the wards the presses for linen are flat on the top and will be a harbour for dust. (I was not able to see the inside of these presses). We note that the infirmary has accommodation for 400 patients, but the nurses' home (which was not open for inspection) only provides bedrooms for eighty nurses. If this is intended to include the nurses for the out-patients' and casualty department, the proportion of nurses to patients is not equal to the requirements.

A special feature in the building is the separate entrance which has been provided for patients' visitors.

The buildings, generally, display an amount of thought and keen appreciation of modern hospital requirements, and the architects are to be congratulated on the completion of their part of the work. It remains for the staff and administrative department to make the best use of the buildings, and we are pleased to note that Miss M'Call Anderson, R.R.C., takes up her duties as matron when the [illegible]pital opens to receive patients early in September.

